# Bis[1,3-bis­(2,4,6-tri­methyl­phen­yl)imidazolium] bis(μ-*cis*-1,2-di­phenyl­ethene-1,2-di­thiol­ato-κ^2^ *S*,*S*′:κ*S*)bis­[(*cis*-1,2-di­phenyl­ethene-1,2-di­thiol­ato-κ^2^ *S*,*S*′)iron(III)] di­methyl­formamide disolvate

**DOI:** 10.1107/S2414314623010830

**Published:** 2023-12-26

**Authors:** Jayaraman Selvakumar, Kuppuswamy Arumugam

**Affiliations:** a Department of Chemistry, Wright State University, 3640 Colonel Glenn Hwy., Dayton, OH 45435, USA; University Koblenz-Landau, Germany

**Keywords:** crystal structure, imidazolium salt, iron bis­(di­thiol­ene) complexes, reduced iron bis­(di­thiol­ene) complexes

## Abstract

The mol­ecular structure of the solvated title salt, (C_21_H_25_N_2_)_2_[Fe_2_(C_14_H_10_S_2_)_4_]·2C_3_H_7_NO reveals that the anion is situated on a crystallographic inversion center in the triclinic space group *P*  . The title compound crystallizes utilizing a network of weak π-stacking inter­actions of phenyl rings pertaining to the di­thiol­ene unit. Moreover, the acidic imidazolium H atoms [N—C(**H**)—N] display non-classical hydrogen-bonding inter­actions of the C—H⋯O type to the oxygen atoms of the *N*,*N*-dimethyl formamide solvent, and hydrogen atoms on the backbone of imidazolium rings display weak C—H⋯S inter­actions with the di­thiol­ene sulfur atoms.

## Abstract 2

The solid-state structural analysis of a reduced iron bis­(di­thiol­ene) dimer reveals that the mol­ecule crystallizes with the center of the Fe_2_S_2_ ring system on a crystallographic center of inversion. The structure shows non-classical hydrogen-bonding inter­actions between imidazolium H atoms and the oxygen atoms of *N*,*N*-dimethyl formamide that co-crystallized together with the title compound.

## Graphical abstract

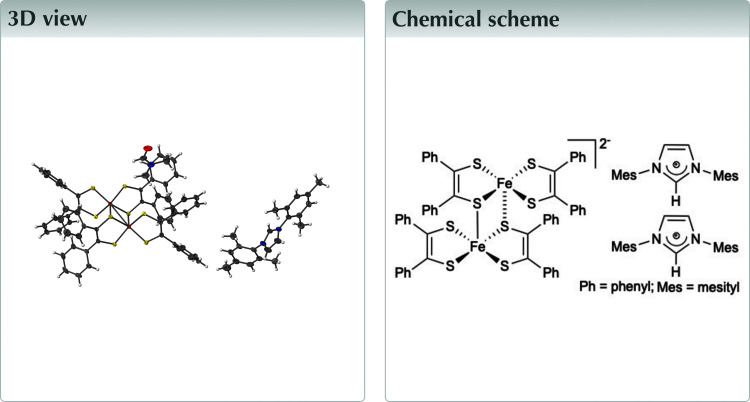

## Structure description

**Figure 1:**
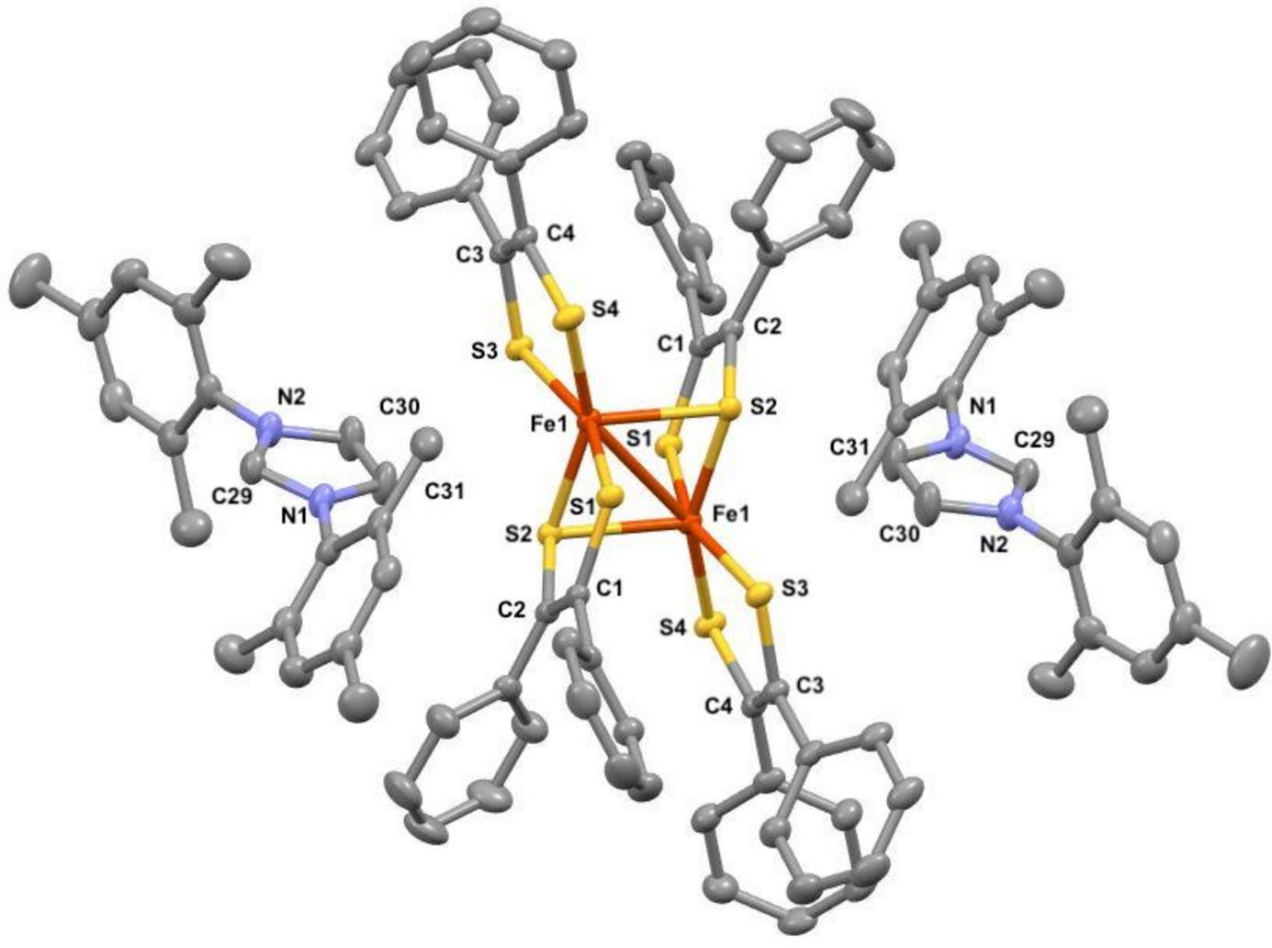
The mol­ecular structure of the cations and the anion in the title compound, with displacement ellipsoids drawn at the 50% probability level. Hydrogen atoms are omitted for clarity. The solvent molecule was omitted for clarity. Only one cation was observed in the asymmetric unit and the other was generated by the inversion center at (−*x*, −*y*, −*z*).

The iron bis­(di­thiol­ene) dimer, [Fe(S_2_C_2_Ph_2_)_2_]_2_ (Schrauzer *et al.*, 1964 ▸), displays a rich electrochemistry and is generally characterized by two successive ligand-based reduction processes pertaining to the di­thiol­ene units (Patra *et al.*, 2006 ▸; Ray *et al.*, 2005 ▸; Yu *et al.*, 2007 ▸). Despite their facile redox processes, the solid-state structure of the chemically reduced species [Fe(S_2_C_2_Ph_2_)_2_]_2_^−^ have not yet been reported. Recently, we reported several five-coordinate iron bis­(di­thiol­ene) complexes with N-heterocyclic carbene ligands [Fe(S_2_C_2_Ph_2_)_2_(NHC)] [NHC = 1,3-bis­(2,4,6-tri­methyl­phen­yl)imidazol-2-yl­idene; Selvakumar *et al.*, 2021 ▸]. Under electrochemical conditions, these complexes undergo two successive one-electron reductions, with the first reduction being reversible whereas the second reduction is irreversible. This irreversibility is attributed to the cleavage of the coordination of the N-heterocyclic carbene ligand to the respective iron atom (Selvakumar *et al.*, 2021 ▸). To isolate and study the solid-state structure of [Fe(S_2_C_2_Ph_2_)_2_NHC]^−^, we reduced the neutral complex [Fe(S_2_C_2_Ph_2_)_2_(NHC)] with a stoichiometric amount of cobaltocene. The reduction process resulted in the decomplexation of NHC and the formation of [Fe(S_2_C_2_Ph_2_)_2_]_2_^2–^ with two NHC[H] cations serving as counter-ions. Herein, we disclose the mol­ecular structure and solid-state structural characteristics of [Fe(S_2_C_2_Ph_2_)_2_]_2_[NHC[H]]_2_ (Fig. 1 ▸).

Since the reductions of the substrate complex are predom­inantly ligand-based (Patra *et al.*, 2006 ▸), the bis­(di­thiol­ene) unit of [(Fe(S_2_C_2_Ph_2_)_2_(NHC)] undergoes a conversion from thienyl radical monoanions (^·^S–C=C–S^−^) to fully reduced 1,2-ene-di­thiol­ate (^−^S–C=C–S^−^). The C—S bond lengths in the title compound [C1—S1 = 1.762 (2) Å, C2—S2 = 1.770 (3) Å, C3—S3 = 1.764 (2) Å, C4—S4 = 1.757 (2) Å] are in agreement with pure C—S single-bond lengths. The C1—C2 [1.353 (3) Å] and C3—C4 [1.352 (3) Å] bond lengths are consistent with a double-bond character. Hence, the inter­pretation of the ligands as 1,2-ene-di­thiol­ates is confirmed.

**Figure 2:**
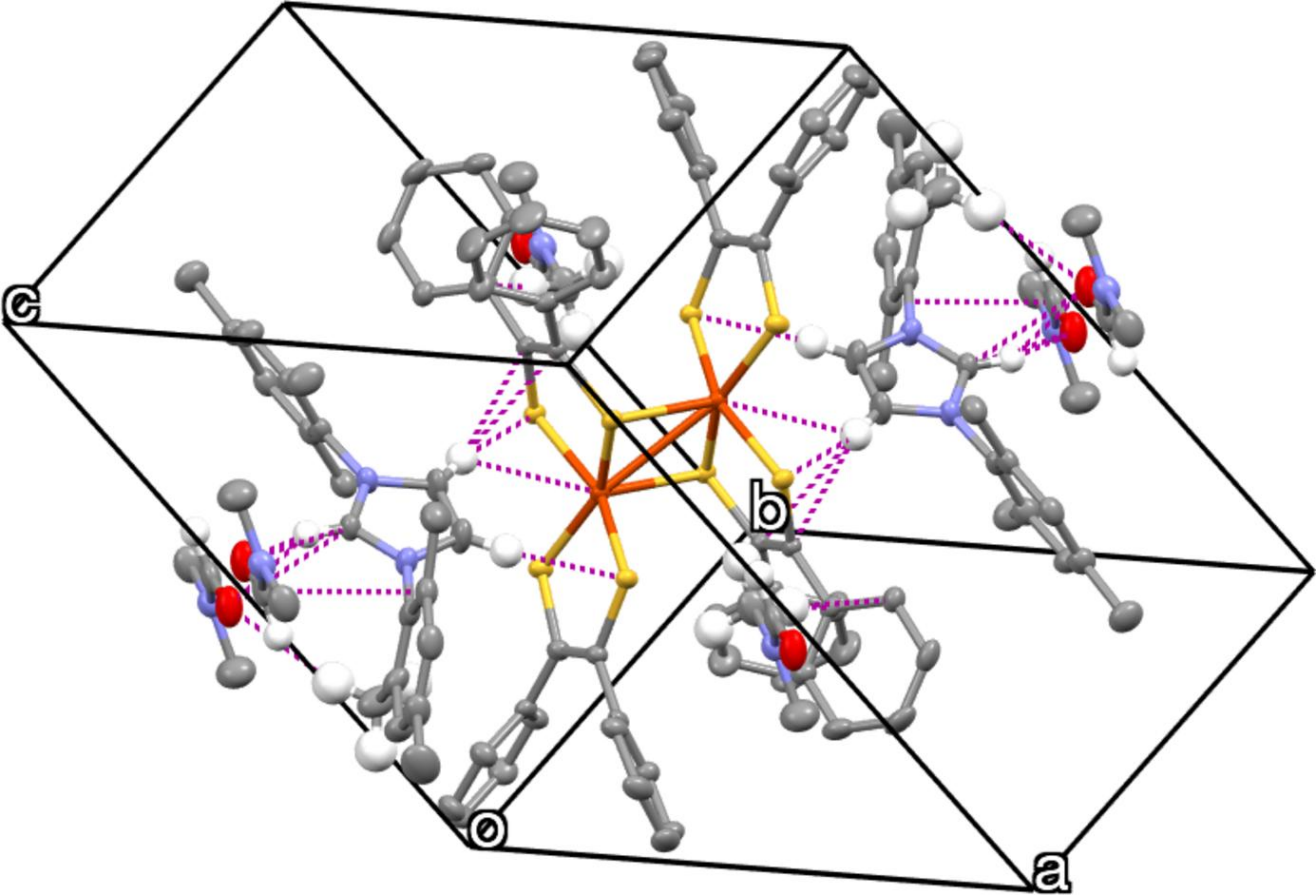
Inter­molecular C—H⋯O and C—H⋯S inter­actions (dotted lines) in the title compound.

**Figure 3:**
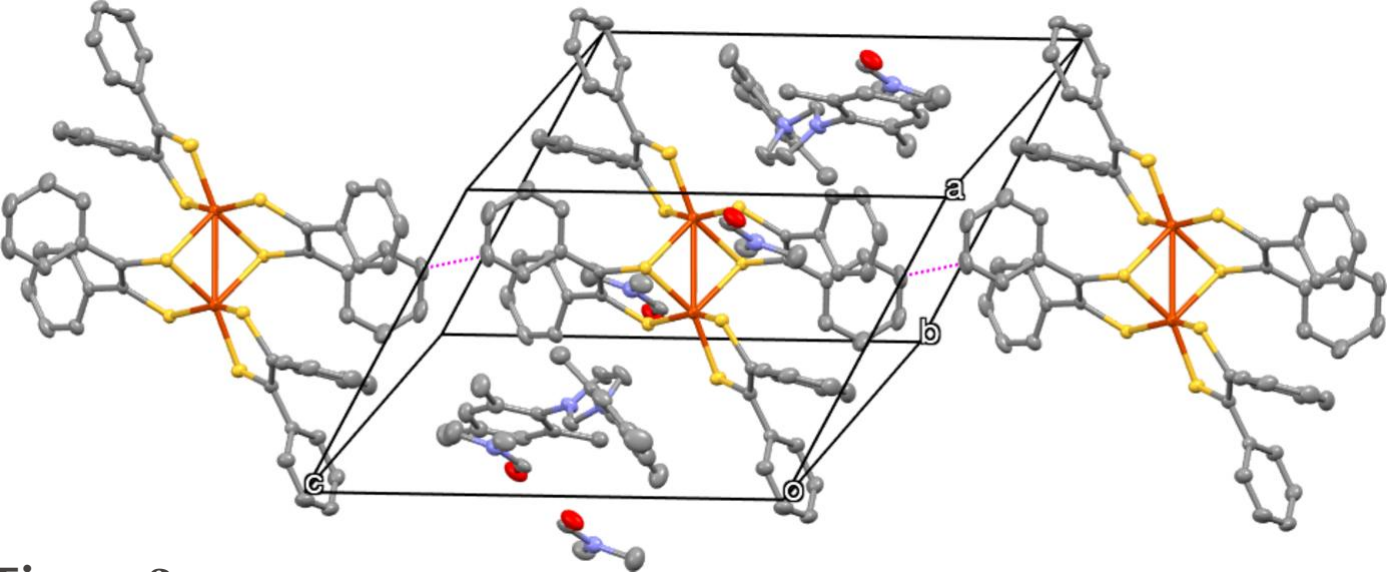
Inter­molecular C⋯C inter­actions of the phenyl rings (dotted lines) in the title compound.

Analysis of the mol­ecular structure revealed no classical hydrogen bonds. However, the presence of non-classical hydrogen-bonding inter­actions involving C—H⋯O and C—H⋯S inter­actions (Fig. 2 ▸) is observed. The C—H⋯O and C—H⋯S inter­actions are detailed in Table 1 ▸. Moreover, the mol­ecules are also inter­connected by inter­molecular C⋯C inter­actions of the phenyl rings pertaining to the di­thiol­ene units (Fig. 3 ▸). All these inter­actions combine to yield a tri-periodic mol­ecular structure.

A CSD structure search for the core [Fe(S_2_C_2_Ph_2_)_2_]_2_^2–^ revealed no hits. However, structurally similar compounds have been reported in the literature, *viz*. [Fe(S_2_C_2_(C_6_H_4_-*p*-OCH_3_)_2_)_2_]_2_^2−^ (Yu *et al.*, 2007 ▸), [Fe(S_2_C_6_H_4_)_2_]^2−^ (Ray *et al.*, 2005 ▸), and [Fe(S_2_C_6_H_3_(*o*-CH_2_CH_3_))_2_]^2−^ (Ray *et al.*, 2005 ▸). The Fe—S, C—S, and C—C bond lengths for [Fe(S_2_C_2_Ph_2_)_2_]_2_[NHC[H]]_2_ are in agreement with those in the reported compounds.

## Synthesis and crystallization

Cobaltocene (Cp_2_Co) (3.8 mg, 0.02 mmol) in 1 ml of *N*,*N*-dimethyl formamide (DMF) was added dropwise to a stirred solution of [(Fe(S_2_C_2_Ph_2_)_2_(NHC)] (17 mg, 0.02 mmol; NHC = 1,3-dimesitylimidazol-2-yl­idene) in 2 ml of DMF. This addition induced a colour change of the solution from dark green to brown. After stirring 30 min at 25°C, the reaction mixture was filtered through a celite plug. The filtrate was subjected to vial in a vial for crystallization according to the vapour diffusion method. Brown plate-like shaped crystals of the title compound [(Fe(S_2_C_2_Ph_2_)_2_)_2_][NHC(**H**)]_2_ were obtained by diffusion of toluene into the brown reaction mixture containing DMF as the solvent (yield: 11 mg, 60%).

## Refinement

Crystal data, data collection and structure refinement details are summarized in Table 2 ▸.

## Supplementary Material

Crystal structure: contains datablock(s) I. DOI: 10.1107/S2414314623010830/im4022sup1.cif

Structure factors: contains datablock(s) I. DOI: 10.1107/S2414314623010830/im4022Isup2.hkl

CCDC reference: 2319850

Additional supporting information:  crystallographic information; 3D view; checkCIF report

## Figures and Tables

**Table 1:** Hydrogen-bond geometry (Å, °)

*D*—H⋯*A*	*D*—H	H⋯*A*	*D*⋯*A*	*D*—H⋯*A*
C29—H29⋯O1	0.93 (1)	2.22 (1)	3.038 (3)	147 (1)
C30—H30⋯O1	0.93 (1)	2.77 (1)	3.593 (3)	148 (1)
C31—H31⋯S1	0.93 (1)	2.87 (1)	3.763 (3)	162 (1)
C49—H49⋯O1	0.96 (1)	2.59 (1)	3.529 (4)	167 (1)

**Table 2:** Experimental details

Crystal data	
Chemical formula	(C_21_H_25_N_2_)_2_[Fe_2_(C_14_H_10_S_82_)_4_]·2C_3_H_7_NO
*M*_r_	919.12
Crystal system, space group	Triclinic, *P* 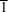
Temperature (K)	100
*a*, *b*, *c* (Å)	12.4019 (14), 14.8205 (19), 14.9762 (18)
α, β, γ (°)	105.936 (4), 113.662 (3), 94.486 (4)
*V* (Å^3^)	2368.2 (5)
*Z*	2
Radiation type	Mo *K*α
μ (mm^−1^)	0.54
Crystal size (mm)	0.57 × 0.28 × 0.2

Data collection	
Diffractometer	Bruker APEXII CCD
No. of measured, independent and observed [*I* ≥ 2u(*I*)] reflections	45150, 8282, 6984
*R*_int_	0.054
(sin θ/λ)_max_ (Å^−1^)	0.595

Refinement	
*R*[*F* ^2^ > 2σ(*F* ^2^)], *wR*(*F* ^2^), *S*	0.033, 0.090, 1.07
No. of reflections	8282
No. of parameters	558
H-atom treatment	H-atom parameters constrained
Δρ_max_, Δρ_min_ (e Å^−3^)	0.54, −0.40

Computer programs: *APEX2* and *SAINT* (Bruker, 2014 ▸), *OLEX2.solve* and *OLEX2.refine* (Bourhis *et al.*, 2015 ▸), and *OLEX2* (Dolomanov *et al.*, 2009 ▸).

## full crystallographic data

### Crystal data

**Table:**

(C_21_H_25_N_2_)_2_[Fe_2_(C_14_H_10_S_82_)_4_]·2C_3_H_7_NO	*Z* = 2
*M**_r_* = 919.12	*F*(000) = 967.9950
Triclinic, *P*1	*D*_x_ = 1.289 Mg m^−^^3^
*a* = 12.4019 (14) Å	Mo *K*α radiation, λ = 0.71073 Å
*b* = 14.8205 (19) Å	Cell parameters from 9889 reflections
*c* = 14.9762 (18) Å	θ = 2.5–24.9°
α = 105.936 (4)°	µ = 0.54 mm^−^^1^
β = 113.662 (3)°	*T* = 100 K
γ = 94.486 (4)°	Prism, black
*V* = 2368.2 (5) Å^3^	0.57 × 0.28 × 0.2 mm

### Data collection

**Table:**

Bruker APEXII CCD diffractometer	*R*_int_ = 0.054
Graphite monochromator	θ_max_ = 25.0°, θ_min_ = 2.8°
φ and ω scans	*h* = −14→14
45150 measured reflections	*k* = −17→17
8282 independent reflections	*l* = −17→17
6984 reflections with *I*≥ 2u(*I*)	

### Refinement

**Table:**

Refinement on *F*^2^	0 restraints
Least-squares matrix: full	88 constraints
*R*[*F*^2^ > 2σ(*F*^2^)] = 0.033	H-atom parameters constrained
*wR*(*F*^2^) = 0.090	*w* = 1/[σ^2^(*F*_o_^2^) + (0.0383*P*)^2^ + 1.4785*P*] where *P* = (*F*_o_^2^ + 2*F*_c_^2^)/3
*S* = 1.07	(Δ/σ)_max_ < 0.001
8282 reflections	Δρ_max_ = 0.54 e Å^−^^3^
558 parameters	Δρ_min_ = −0.40 e Å^−^^3^

### Special details

**Table:**

Refinement. Aromatic C–H H atoms were added using the riding-model approximation with C–H bond lengths of 0.95 Å with *U*_iso_ (H) = 1.2 *U*_eq_(C_ar_H). Methyl (CH_3_) H atoms were treated as a rotating group and added using the riding-model approximation to the carbon atom to which they are attached. Methyl H atoms were fixed at a distance of 0.98 Å with *U*_iso_ (H) = 1.5 *U*_eq_(CH_3_). The title compound co-crystallized with one solvent molecule (N, *N*-dimethyl formamide) per asymmetric unit of the unit cell.

### Fractional atomic coordinates and isotropic or equivalent isotropic displacement parameters (Å^2^)

**Table:**

	*x*	*y*	*z*	*U*_iso_*/*U*_eq_	
Fe1	0.62110 (3)	0.564448 (19)	0.55925 (2)	0.01723 (9)	
N1	−0.17750 (16)	−0.23003 (12)	−0.57291 (14)	0.0258 (4)	
S2	0.47668 (4)	0.56334 (3)	0.40931 (4)	0.01803 (12)	
S1	0.70483 (4)	0.47741 (3)	0.46806 (4)	0.01933 (12)	
S3	0.57529 (4)	0.69570 (3)	0.63988 (4)	0.01979 (12)	
S4	0.80666 (5)	0.61643 (4)	0.68790 (4)	0.02292 (12)	
O1	0.89705 (16)	0.04702 (13)	0.41971 (15)	0.0425 (4)	
C32	−0.12009 (19)	−0.28038 (14)	−0.63316 (17)	0.0250 (5)	
C41	−0.2576 (2)	−0.02146 (15)	−0.45253 (18)	0.0272 (5)	
N3	0.76879 (17)	0.14028 (13)	0.35900 (15)	0.0304 (4)	
C29	−0.16751 (19)	−0.13524 (15)	−0.54035 (17)	0.0256 (5)	
H29	−0.12006 (19)	−0.09024 (15)	−0.54958 (17)	0.0307 (6)*	
C31	−0.2557 (2)	−0.27257 (17)	−0.5440 (2)	0.0356 (6)	
H31	−0.2783 (2)	−0.33792 (17)	−0.5570 (2)	0.0428 (7)*	
C30	−0.2928 (2)	−0.20136 (17)	−0.4936 (2)	0.0381 (6)	
H30	−0.3458 (2)	−0.20830 (17)	−0.4650 (2)	0.0458 (7)*	
C51	0.7468 (3)	0.0959 (2)	0.2527 (2)	0.0532 (8)	
H51a	0.7881 (16)	0.1389 (6)	0.2334 (5)	0.0799 (11)*	
H51b	0.7758 (17)	0.0374 (8)	0.2456 (4)	0.0799 (11)*	
H51c	0.6618 (3)	0.0819 (14)	0.2086 (3)	0.0799 (11)*	
C28	0.7159 (2)	0.83634 (16)	0.93701 (17)	0.0301 (5)	
H28	0.7528 (2)	0.78961 (16)	0.96034 (17)	0.0362 (6)*	
C47	−0.4322 (2)	−0.0556 (2)	−0.6289 (2)	0.0439 (6)	
H47a	−0.5065 (8)	−0.0346 (9)	−0.6550 (5)	0.0658 (10)*	
H47b	−0.3904 (7)	−0.0505 (11)	−0.6697 (3)	0.0658 (10)*	
H47c	−0.4488 (14)	−0.1212 (3)	−0.6326 (3)	0.0658 (10)*	
C20	1.1390 (2)	0.83725 (18)	1.05341 (17)	0.0335 (6)	
H20	1.2120 (2)	0.86338 (18)	1.11234 (17)	0.0402 (7)*	
C3	0.70124 (19)	0.75091 (14)	0.76107 (15)	0.0216 (4)	
C38	−0.2435 (3)	−0.2183 (2)	−0.7778 (2)	0.0441 (7)	
H38a	−0.3109 (8)	−0.2294 (11)	−0.7631 (13)	0.0662 (10)*	
H38b	−0.2070 (5)	−0.1510 (2)	−0.7483 (11)	0.0662 (10)*	
H38c	−0.2706 (13)	−0.2388 (10)	−0.8514 (3)	0.0662 (10)*	
C46	−0.1792 (2)	0.03704 (17)	−0.35228 (18)	0.0311 (5)	
C2	0.52106 (18)	0.50821 (14)	0.31254 (15)	0.0186 (4)	
C6	0.7759 (2)	0.41881 (16)	0.28058 (17)	0.0270 (5)	
H6	0.8338 (2)	0.46730 (16)	0.33885 (17)	0.0324 (6)*	
C12	0.5064 (2)	0.55707 (18)	0.16129 (18)	0.0341 (5)	
H12	0.5901 (2)	0.57460 (18)	0.19153 (18)	0.0409 (7)*	
C42	−0.3549 (2)	0.00652 (17)	−0.51813 (19)	0.0307 (5)	
C40	0.0357 (3)	−0.43837 (19)	−0.8171 (2)	0.0436 (7)	
H40a	0.1087 (9)	−0.4529 (12)	−0.7738 (2)	0.0654 (10)*	
H40b	−0.0216 (7)	−0.4971 (6)	−0.8638 (10)	0.0654 (10)*	
H40c	0.0528 (16)	−0.4022 (6)	−0.8559 (11)	0.0654 (10)*	
C4	0.80308 (19)	0.71629 (14)	0.78183 (15)	0.0220 (4)	
C48	−0.3211 (3)	0.2570 (2)	−0.3390 (3)	0.0611 (9)	
H48a	−0.2748 (16)	0.3033 (3)	−0.3510 (16)	0.0917 (14)*	
H48b	−0.4052 (5)	0.2563 (5)	−0.3746 (13)	0.0917 (14)*	
H48c	−0.2977 (19)	0.2740 (7)	−0.2661 (4)	0.0917 (14)*	
C45	−0.2011 (2)	0.12767 (17)	−0.31768 (19)	0.0364 (6)	
H45	−0.1490 (2)	0.16899 (17)	−0.25154 (19)	0.0437 (7)*	
C34	0.0128 (2)	−0.38324 (16)	−0.65194 (18)	0.0294 (5)	
H34	0.0680 (2)	−0.41932 (16)	−0.62582 (18)	0.0353 (6)*	
C13	0.4389 (3)	0.5702 (2)	0.0680 (2)	0.0465 (7)	
H13	0.4776 (3)	0.5968 (2)	0.0364 (2)	0.0559 (8)*	
C5	0.65553 (19)	0.40751 (14)	0.26261 (15)	0.0205 (4)	
C1	0.61786 (18)	0.46720 (14)	0.33805 (15)	0.0190 (4)	
C36	−0.0978 (2)	−0.32501 (17)	−0.78864 (18)	0.0341 (5)	
H36	−0.1167 (2)	−0.32200 (17)	−0.85432 (18)	0.0409 (7)*	
C39	−0.0080 (2)	−0.34176 (17)	−0.48534 (18)	0.0333 (5)	
H39a	0.0048 (14)	−0.2794 (3)	−0.4369 (3)	0.0499 (8)*	
H39b	−0.0732 (7)	−0.3844 (9)	−0.4890 (3)	0.0499 (8)*	
H39c	0.0640 (8)	−0.3664 (11)	−0.4630 (6)	0.0499 (8)*	
N2	−0.23739 (17)	−0.11610 (13)	−0.49220 (15)	0.0277 (4)	
C43	−0.3738 (2)	0.09706 (17)	−0.4782 (2)	0.0364 (6)	
H43	−0.4391 (2)	0.11715 (17)	−0.5195 (2)	0.0437 (7)*	
C22	0.9777 (2)	0.70262 (16)	0.93387 (17)	0.0283 (5)	
H22	0.9432 (2)	0.63787 (16)	0.91280 (17)	0.0339 (6)*	
C19	1.0825 (2)	0.89456 (17)	0.99812 (18)	0.0351 (6)	
H19	1.1177 (2)	0.95922 (17)	1.01954 (18)	0.0421 (7)*	
C44	−0.2983 (2)	0.15838 (17)	−0.3789 (2)	0.0375 (6)	
C16	0.3247 (2)	0.49265 (18)	0.16272 (18)	0.0330 (5)	
H16	0.2850 (2)	0.46698 (18)	0.19417 (18)	0.0396 (6)*	
C10	0.5705 (2)	0.33425 (15)	0.17443 (16)	0.0270 (5)	
H10	0.4896 (2)	0.32567 (15)	0.16071 (16)	0.0324 (6)*	
C21	1.0869 (2)	0.74125 (18)	1.02099 (18)	0.0337 (5)	
H21	1.1251 (2)	0.70247 (18)	1.05773 (18)	0.0405 (6)*	
C26	0.6386 (2)	0.97932 (17)	0.97090 (19)	0.0375 (6)	
H26	0.6234 (2)	1.02789 (17)	1.01591 (19)	0.0451 (7)*	
C17	0.91867 (19)	0.75881 (15)	0.87719 (15)	0.0233 (5)	
C35	−0.0160 (2)	−0.38001 (16)	−0.75048 (18)	0.0310 (5)	
C25	0.6059 (2)	0.97638 (16)	0.87041 (18)	0.0336 (6)	
H25	0.5689 (2)	1.02335 (16)	0.84773 (18)	0.0404 (7)*	
C9	0.6060 (2)	0.27418 (17)	0.10719 (18)	0.0355 (6)	
H9	0.5487 (2)	0.22552 (17)	0.04871 (18)	0.0426 (7)*	
C23	0.68333 (18)	0.83176 (14)	0.83491 (16)	0.0226 (5)	
C24	0.62802 (19)	0.90339 (15)	0.80279 (17)	0.0280 (5)	
H24	0.60569 (19)	0.90222 (15)	0.73525 (17)	0.0336 (6)*	
C18	0.9734 (2)	0.85541 (16)	0.91072 (17)	0.0301 (5)	
H18	0.9360 (2)	0.89431 (16)	0.87377 (17)	0.0361 (6)*	
C52	0.7088 (2)	0.21868 (18)	0.3806 (2)	0.0417 (6)	
H52a	0.7356 (12)	0.2458 (9)	0.4539 (2)	0.0625 (10)*	
H52b	0.7282 (13)	0.2673 (6)	0.3550 (13)	0.0625 (10)*	
H52c	0.6232 (2)	0.1945 (3)	0.3471 (12)	0.0625 (10)*	
C15	0.2587 (2)	0.5054 (2)	0.06950 (19)	0.0442 (7)	
H15	0.1751 (2)	0.4874 (2)	0.03849 (19)	0.0531 (8)*	
C27	0.6942 (2)	0.90956 (17)	1.00431 (19)	0.0372 (6)	
H27	0.7172 (2)	0.91171 (17)	1.07223 (19)	0.0446 (7)*	
C49	−0.0740 (3)	0.0049 (2)	−0.2849 (2)	0.0490 (7)	
H49a	−0.0160 (9)	0.0002 (14)	−0.3123 (9)	0.0735 (11)*	
H49b	−0.0375 (11)	0.0506 (8)	−0.2160 (4)	0.0735 (11)*	
H49c	−0.1014 (4)	−0.0568 (7)	−0.2832 (12)	0.0735 (11)*	
C7	0.8108 (2)	0.35869 (18)	0.21271 (19)	0.0349 (6)	
H7	0.8914 (2)	0.36738 (18)	0.22544 (19)	0.0419 (7)*	
C8	0.7261 (3)	0.28626 (18)	0.12664 (19)	0.0390 (6)	
H8	0.7495 (3)	0.24550 (18)	0.08161 (19)	0.0468 (7)*	
C50	0.8429 (2)	0.11203 (16)	0.43295 (19)	0.0298 (5)	
H50	0.8552 (2)	0.14359 (16)	0.50027 (19)	0.0358 (6)*	
C14	0.3151 (3)	0.5444 (2)	0.0218 (2)	0.0477 (7)	
H14	0.2700 (3)	0.5532 (2)	−0.0407 (2)	0.0572 (8)*	
C11	0.4498 (2)	0.51773 (15)	0.21005 (16)	0.0233 (5)	
C33	−0.03867 (19)	−0.33393 (15)	−0.59061 (17)	0.0257 (5)	
C37	−0.1518 (2)	−0.27453 (16)	−0.73156 (18)	0.0295 (5)	

### Atomic displacement parameters (Å^2^)

**Table:**

	*U*^11^	*U*^22^	*U*^33^	*U*^12^	*U*^13^	*U*^23^
Fe1	0.01874 (16)	0.01510 (15)	0.01784 (15)	0.00388 (11)	0.01013 (12)	0.00228 (11)
N1	0.0266 (10)	0.0184 (9)	0.0361 (10)	0.0042 (7)	0.0188 (9)	0.0071 (8)
S2	0.0202 (3)	0.0168 (2)	0.0188 (2)	0.00595 (19)	0.0109 (2)	0.00447 (19)
S1	0.0201 (3)	0.0190 (2)	0.0193 (2)	0.0066 (2)	0.0107 (2)	0.00334 (19)
S3	0.0192 (3)	0.0172 (2)	0.0204 (2)	0.0046 (2)	0.0095 (2)	0.00126 (19)
S4	0.0200 (3)	0.0211 (3)	0.0221 (3)	0.0055 (2)	0.0088 (2)	−0.0005 (2)
O1	0.0375 (10)	0.0379 (10)	0.0721 (13)	0.0193 (8)	0.0321 (10)	0.0327 (9)
C32	0.0252 (11)	0.0165 (10)	0.0333 (12)	0.0024 (9)	0.0168 (10)	0.0030 (9)
C41	0.0343 (13)	0.0220 (11)	0.0382 (13)	0.0103 (9)	0.0264 (11)	0.0123 (10)
N3	0.0292 (10)	0.0255 (10)	0.0377 (11)	0.0095 (8)	0.0142 (9)	0.0121 (8)
C29	0.0265 (12)	0.0206 (11)	0.0348 (12)	0.0048 (9)	0.0185 (10)	0.0093 (9)
C31	0.0407 (14)	0.0218 (12)	0.0572 (16)	0.0049 (10)	0.0332 (13)	0.0147 (11)
C30	0.0466 (15)	0.0269 (12)	0.0603 (16)	0.0087 (11)	0.0397 (14)	0.0183 (12)
C51	0.063 (2)	0.0543 (18)	0.0410 (15)	0.0170 (15)	0.0214 (15)	0.0153 (13)
C28	0.0368 (13)	0.0245 (11)	0.0301 (12)	0.0055 (10)	0.0190 (11)	0.0045 (9)
C47	0.0357 (15)	0.0482 (16)	0.0436 (15)	0.0071 (12)	0.0162 (12)	0.0117 (13)
C20	0.0230 (12)	0.0403 (14)	0.0251 (11)	−0.0008 (10)	0.0074 (10)	0.0003 (10)
C3	0.0236 (11)	0.0184 (10)	0.0200 (10)	0.0014 (8)	0.0104 (9)	0.0021 (8)
C38	0.0530 (17)	0.0453 (16)	0.0445 (15)	0.0284 (14)	0.0245 (13)	0.0209 (13)
C46	0.0387 (14)	0.0327 (12)	0.0341 (12)	0.0147 (11)	0.0251 (11)	0.0133 (10)
C2	0.0207 (11)	0.0162 (10)	0.0196 (10)	0.0026 (8)	0.0118 (8)	0.0027 (8)
C6	0.0289 (12)	0.0257 (11)	0.0306 (12)	0.0055 (9)	0.0183 (10)	0.0076 (9)
C12	0.0364 (14)	0.0402 (14)	0.0336 (13)	0.0087 (11)	0.0197 (11)	0.0174 (11)
C42	0.0294 (12)	0.0303 (12)	0.0399 (13)	0.0063 (10)	0.0208 (11)	0.0142 (10)
C40	0.0511 (17)	0.0403 (15)	0.0477 (16)	0.0212 (13)	0.0301 (14)	0.0113 (12)
C4	0.0250 (11)	0.0169 (10)	0.0222 (10)	0.0032 (8)	0.0112 (9)	0.0031 (8)
C48	0.060 (2)	0.0322 (15)	0.093 (3)	0.0219 (14)	0.0407 (19)	0.0109 (15)
C45	0.0407 (14)	0.0328 (13)	0.0369 (13)	0.0092 (11)	0.0227 (12)	0.0044 (11)
C34	0.0239 (12)	0.0237 (11)	0.0402 (13)	0.0070 (9)	0.0138 (10)	0.0100 (10)
C13	0.0589 (19)	0.0588 (18)	0.0403 (15)	0.0160 (15)	0.0301 (14)	0.0301 (13)
C5	0.0273 (11)	0.0190 (10)	0.0214 (10)	0.0079 (9)	0.0152 (9)	0.0081 (8)
C1	0.0231 (11)	0.0162 (10)	0.0183 (10)	0.0020 (8)	0.0121 (9)	0.0029 (8)
C36	0.0438 (15)	0.0310 (12)	0.0313 (12)	0.0124 (11)	0.0205 (11)	0.0090 (10)
C39	0.0302 (13)	0.0319 (13)	0.0360 (13)	0.0041 (10)	0.0134 (11)	0.0114 (10)
N2	0.0329 (11)	0.0214 (9)	0.0383 (11)	0.0080 (8)	0.0243 (9)	0.0103 (8)
C43	0.0311 (13)	0.0354 (13)	0.0552 (16)	0.0168 (11)	0.0243 (12)	0.0229 (12)
C22	0.0297 (12)	0.0213 (11)	0.0274 (11)	0.0031 (9)	0.0109 (10)	0.0020 (9)
C19	0.0301 (13)	0.0262 (12)	0.0379 (13)	−0.0041 (10)	0.0130 (11)	0.0006 (10)
C44	0.0416 (15)	0.0259 (12)	0.0557 (16)	0.0133 (11)	0.0307 (13)	0.0135 (11)
C16	0.0303 (13)	0.0434 (14)	0.0302 (12)	0.0110 (11)	0.0152 (10)	0.0157 (11)
C10	0.0320 (13)	0.0243 (11)	0.0249 (11)	0.0058 (9)	0.0151 (10)	0.0048 (9)
C21	0.0316 (13)	0.0378 (13)	0.0288 (12)	0.0089 (11)	0.0103 (10)	0.0111 (10)
C26	0.0376 (14)	0.0305 (13)	0.0391 (14)	0.0056 (11)	0.0233 (12)	−0.0057 (11)
C17	0.0219 (11)	0.0242 (11)	0.0216 (10)	0.0036 (9)	0.0127 (9)	−0.0002 (9)
C35	0.0319 (13)	0.0240 (11)	0.0375 (13)	0.0083 (10)	0.0186 (11)	0.0055 (10)
C25	0.0280 (12)	0.0238 (12)	0.0385 (13)	0.0075 (10)	0.0119 (11)	−0.0016 (10)
C9	0.0497 (16)	0.0278 (12)	0.0263 (12)	0.0069 (11)	0.0201 (11)	0.0005 (10)
C23	0.0188 (11)	0.0170 (10)	0.0262 (11)	−0.0010 (8)	0.0112 (9)	−0.0021 (8)
C24	0.0241 (12)	0.0237 (11)	0.0270 (11)	0.0040 (9)	0.0085 (10)	−0.0002 (9)
C18	0.0290 (12)	0.0244 (12)	0.0317 (12)	0.0033 (9)	0.0113 (10)	0.0057 (10)
C52	0.0362 (14)	0.0315 (13)	0.0637 (18)	0.0177 (11)	0.0236 (13)	0.0201 (13)
C15	0.0338 (14)	0.0660 (18)	0.0303 (13)	0.0144 (13)	0.0084 (11)	0.0200 (13)
C27	0.0472 (15)	0.0333 (13)	0.0296 (12)	0.0049 (11)	0.0224 (12)	0.0010 (10)
C49	0.0555 (18)	0.0579 (18)	0.0389 (15)	0.0297 (15)	0.0214 (14)	0.0184 (13)
C7	0.0389 (14)	0.0393 (14)	0.0459 (14)	0.0170 (11)	0.0329 (12)	0.0187 (12)
C8	0.0612 (18)	0.0340 (13)	0.0395 (14)	0.0207 (12)	0.0386 (13)	0.0106 (11)
C50	0.0238 (12)	0.0261 (12)	0.0441 (14)	0.0026 (10)	0.0171 (11)	0.0158 (10)
C14	0.0560 (18)	0.0659 (19)	0.0278 (13)	0.0238 (15)	0.0167 (13)	0.0256 (13)
C11	0.0306 (12)	0.0212 (10)	0.0210 (10)	0.0091 (9)	0.0146 (9)	0.0053 (8)
C33	0.0231 (11)	0.0181 (10)	0.0342 (12)	0.0012 (9)	0.0125 (10)	0.0071 (9)
C37	0.0311 (13)	0.0224 (11)	0.0366 (13)	0.0102 (9)	0.0160 (10)	0.0093 (10)

### Geometric parameters (Å, º)

**Table:**

Fe1—Fe1^i^	2.9636 (6)	C40—C35	1.510 (3)
Fe1—S2^i^	2.4224 (6)	C4—C17	1.488 (3)
Fe1—S2	2.2355 (6)	C48—H48a	0.9600
Fe1—S1	2.2334 (6)	C48—H48b	0.9600
Fe1—S3	2.2357 (6)	C48—H48c	0.9600
Fe1—S4	2.2249 (6)	C48—C44	1.515 (3)
N1—C32	1.452 (3)	C45—H45	0.9300
N1—C29	1.332 (3)	C45—C44	1.389 (4)
N1—C31	1.384 (3)	C34—H34	0.9300
S2—C2	1.7695 (19)	C34—C35	1.388 (3)
S1—C1	1.762 (2)	C34—C33	1.400 (3)
S3—C3	1.764 (2)	C13—H13	0.9300
S4—C4	1.757 (2)	C13—C14	1.376 (4)
O1—C50	1.234 (3)	C5—C1	1.489 (3)
C32—C33	1.394 (3)	C5—C10	1.397 (3)
C32—C37	1.394 (3)	C36—H36	0.9300
C41—C46	1.390 (3)	C36—C35	1.392 (3)
C41—C42	1.394 (3)	C36—C37	1.388 (3)
C41—N2	1.449 (3)	C39—H39a	0.9600
N3—C51	1.445 (3)	C39—H39b	0.9600
N3—C52	1.459 (3)	C39—H39c	0.9600
N3—C50	1.321 (3)	C39—C33	1.507 (3)
C29—H29	0.9300	C43—H43	0.9300
C29—N2	1.334 (3)	C43—C44	1.386 (4)
C31—H31	0.9300	C22—H22	0.9300
C31—C30	1.347 (3)	C22—C21	1.383 (3)
C30—H30	0.9300	C22—C17	1.391 (3)
C30—N2	1.381 (3)	C19—H19	0.9300
C51—H51a	0.9600	C19—C18	1.386 (3)
C51—H51b	0.9600	C16—H16	0.9300
C51—H51c	0.9600	C16—C15	1.381 (3)
C28—H28	0.9300	C16—C11	1.388 (3)
C28—C23	1.396 (3)	C10—H10	0.9300
C28—C27	1.389 (3)	C10—C9	1.387 (3)
C47—H47a	0.9600	C21—H21	0.9300
C47—H47b	0.9600	C26—H26	0.9300
C47—H47c	0.9600	C26—C25	1.379 (4)
C47—C42	1.505 (3)	C26—C27	1.383 (4)
C20—H20	0.9300	C17—C18	1.394 (3)
C20—C19	1.383 (4)	C25—H25	0.9300
C20—C21	1.380 (3)	C25—C24	1.392 (3)
C3—C4	1.352 (3)	C9—H9	0.9300
C3—C23	1.490 (3)	C9—C8	1.385 (4)
C38—H38a	0.9600	C23—C24	1.399 (3)
C38—H38b	0.9600	C24—H24	0.9300
C38—H38c	0.9600	C18—H18	0.9300
C38—C37	1.517 (3)	C52—H52a	0.9600
C46—C45	1.391 (3)	C52—H52b	0.9600
C46—C49	1.501 (4)	C52—H52c	0.9600
C2—C1	1.353 (3)	C15—H15	0.9300
C2—C11	1.485 (3)	C15—C14	1.377 (4)
C6—H6	0.9300	C27—H27	0.9300
C6—C5	1.394 (3)	C49—H49a	0.9600
C6—C7	1.389 (3)	C49—H49b	0.9600
C12—H12	0.9300	C49—H49c	0.9600
C12—C13	1.389 (3)	C7—H7	0.9300
C12—C11	1.396 (3)	C7—C8	1.376 (4)
C42—C43	1.390 (3)	C8—H8	0.9300
C40—H40a	0.9600	C50—H50	0.9300
C40—H40b	0.9600	C14—H14	0.9300
C40—H40c	0.9600		
			
S1—Fe1—S2^i^	98.85 (2)	C10—C5—C6	118.36 (19)
S1—Fe1—S2	87.69 (2)	C10—C5—C1	119.97 (19)
S3—Fe1—S2	88.78 (2)	C2—C1—S1	119.93 (15)
S3—Fe1—S2^i^	103.39 (2)	C5—C1—S1	115.20 (15)
S3—Fe1—S1	157.74 (2)	C5—C1—C2	124.79 (18)
S4—Fe1—S2	152.39 (2)	C35—C36—H36	119.02 (14)
S4—Fe1—S2^i^	106.42 (2)	C37—C36—H36	119.02 (14)
S4—Fe1—S1	85.80 (2)	C37—C36—C35	122.0 (2)
S4—Fe1—S3	87.22 (2)	H39b—C39—H39a	109.5
C29—N1—C32	125.31 (18)	H39c—C39—H39a	109.5
C31—N1—C32	125.76 (18)	H39c—C39—H39b	109.5
C31—N1—C29	108.87 (18)	C33—C39—H39a	109.5
C2—S2—Fe1^i^	107.03 (7)	C33—C39—H39b	109.5
C2—S2—Fe1	106.33 (7)	C33—C39—H39c	109.5
C1—S1—Fe1	106.41 (7)	C29—N2—C41	124.67 (18)
C3—S3—Fe1	106.36 (7)	C30—N2—C41	126.34 (18)
C4—S4—Fe1	106.94 (7)	C30—N2—C29	108.84 (18)
C33—C32—N1	118.2 (2)	H43—C43—C42	118.91 (15)
C37—C32—N1	118.17 (19)	C44—C43—C42	122.2 (2)
C37—C32—C33	123.6 (2)	C44—C43—H43	118.91 (14)
C42—C41—C46	123.3 (2)	C21—C22—H22	119.38 (14)
N2—C41—C46	118.9 (2)	C17—C22—H22	119.38 (12)
N2—C41—C42	117.8 (2)	C17—C22—C21	121.2 (2)
C52—N3—C51	117.2 (2)	H19—C19—C20	120.08 (13)
C50—N3—C51	120.7 (2)	C18—C19—C20	119.8 (2)
C50—N3—C52	122.1 (2)	C18—C19—H19	120.08 (14)
H29—C29—N1	125.93 (12)	C45—C44—C48	121.1 (2)
N2—C29—N1	108.13 (18)	C43—C44—C48	120.4 (2)
N2—C29—H29	125.93 (12)	C43—C44—C45	118.5 (2)
H31—C31—N1	126.51 (12)	C15—C16—H16	119.62 (16)
C30—C31—N1	107.0 (2)	C11—C16—H16	119.62 (13)
C30—C31—H31	126.51 (14)	C11—C16—C15	120.8 (2)
H30—C30—C31	126.41 (14)	H10—C10—C5	119.80 (12)
N2—C30—C31	107.2 (2)	C9—C10—C5	120.4 (2)
N2—C30—H30	126.41 (12)	C9—C10—H10	119.80 (14)
H51a—C51—N3	109.5	C22—C21—C20	120.0 (2)
H51b—C51—N3	109.5	H21—C21—C20	119.98 (14)
H51b—C51—H51a	109.5	H21—C21—C22	119.98 (14)
H51c—C51—N3	109.5	C25—C26—H26	120.21 (14)
H51c—C51—H51a	109.5	C27—C26—H26	120.21 (14)
H51c—C51—H51b	109.5	C27—C26—C25	119.6 (2)
C23—C28—H28	119.47 (13)	C22—C17—C4	121.03 (19)
C27—C28—H28	119.47 (15)	C18—C17—C4	121.1 (2)
C27—C28—C23	121.1 (2)	C18—C17—C22	117.8 (2)
H47b—C47—H47a	109.5	C34—C35—C40	120.8 (2)
H47c—C47—H47a	109.5	C36—C35—C40	120.5 (2)
H47c—C47—H47b	109.5	C36—C35—C34	118.6 (2)
C42—C47—H47a	109.5	H25—C25—C26	119.87 (14)
C42—C47—H47b	109.5	C24—C25—C26	120.3 (2)
C42—C47—H47c	109.5	C24—C25—H25	119.87 (15)
C19—C20—H20	120.06 (13)	H9—C9—C10	119.81 (14)
C21—C20—H20	120.06 (14)	C8—C9—C10	120.4 (2)
C21—C20—C19	119.9 (2)	C8—C9—H9	119.81 (13)
C4—C3—S3	119.37 (15)	C3—C23—C28	121.3 (2)
C23—C3—S3	115.50 (15)	C24—C23—C28	117.72 (19)
C23—C3—C4	125.09 (18)	C24—C23—C3	120.96 (19)
H38b—C38—H38a	109.5	C23—C24—C25	121.0 (2)
H38c—C38—H38a	109.5	H24—C24—C25	119.50 (15)
H38c—C38—H38b	109.5	H24—C24—C23	119.50 (12)
C37—C38—H38a	109.5	C17—C18—C19	121.2 (2)
C37—C38—H38b	109.5	H18—C18—C19	119.40 (14)
C37—C38—H38c	109.5	H18—C18—C17	119.40 (13)
C45—C46—C41	117.0 (2)	H52a—C52—N3	109.5
C49—C46—C41	121.6 (2)	H52b—C52—N3	109.5
C49—C46—C45	121.3 (2)	H52b—C52—H52a	109.5
C1—C2—S2	119.10 (15)	H52c—C52—N3	109.5
C11—C2—S2	114.65 (14)	H52c—C52—H52a	109.5
C11—C2—C1	126.13 (18)	H52c—C52—H52b	109.5
C5—C6—H6	119.52 (12)	H15—C15—C16	119.55 (16)
C7—C6—H6	119.52 (14)	C14—C15—C16	120.9 (3)
C7—C6—C5	121.0 (2)	C14—C15—H15	119.55 (16)
C13—C12—H12	119.74 (16)	C26—C27—C28	120.4 (2)
C11—C12—H12	119.74 (14)	H27—C27—C28	119.81 (15)
C11—C12—C13	120.5 (2)	H27—C27—C26	119.81 (14)
C47—C42—C41	121.9 (2)	H49a—C49—C46	109.5
C43—C42—C41	116.9 (2)	H49b—C49—C46	109.5
C43—C42—C47	121.2 (2)	H49b—C49—H49a	109.5
H40b—C40—H40a	109.5	H49c—C49—C46	109.5
H40c—C40—H40a	109.5	H49c—C49—H49a	109.5
H40c—C40—H40b	109.5	H49c—C49—H49b	109.5
C35—C40—H40a	109.5	H7—C7—C6	120.00 (14)
C35—C40—H40b	109.5	C8—C7—C6	120.0 (2)
C35—C40—H40c	109.5	C8—C7—H7	120.00 (14)
C3—C4—S4	119.26 (15)	C7—C8—C9	119.9 (2)
C17—C4—S4	115.04 (15)	H8—C8—C9	120.05 (13)
C17—C4—C3	125.60 (18)	H8—C8—C7	120.05 (14)
H48b—C48—H48a	109.5	N3—C50—O1	125.1 (2)
H48c—C48—H48a	109.5	H50—C50—O1	117.45 (15)
H48c—C48—H48b	109.5	H50—C50—N3	117.45 (14)
C44—C48—H48a	109.5	C15—C14—C13	119.1 (2)
C44—C48—H48b	109.5	H14—C14—C13	120.43 (15)
C44—C48—H48c	109.5	H14—C14—C15	120.43 (16)
H45—C45—C46	118.98 (15)	C12—C11—C2	120.8 (2)
C44—C45—C46	122.0 (2)	C16—C11—C2	121.00 (19)
C44—C45—H45	118.98 (14)	C16—C11—C12	118.1 (2)
C35—C34—H34	118.95 (13)	C34—C33—C32	116.6 (2)
C33—C34—H34	118.95 (13)	C39—C33—C32	122.9 (2)
C33—C34—C35	122.1 (2)	C39—C33—C34	120.5 (2)
H13—C13—C12	119.71 (16)	C38—C37—C32	122.8 (2)
C14—C13—C12	120.6 (2)	C36—C37—C32	117.1 (2)
C14—C13—H13	119.71 (15)	C36—C37—C38	120.0 (2)
C1—C5—C6	121.56 (18)		

Symmetry code: (i) −*x*+1, −*y*+1, −*z*+1.

### Hydrogen-bond geometry (Å, º)

**Table:**

*D*—H···*A*	*D*—H	H···*A*	*D*···*A*	*D*—H···*A*
C29—H29···O1	0.93 (1)	2.22 (1)	3.038 (3)	147 (1)
C30—H30···O1	0.93 (1)	2.77 (1)	3.593 (3)	148 (1)
C31—H31···S1	0.93 (1)	2.87 (1)	3.763 (3)	162 (1)
C49—H49···O1	0.96 (1)	2.59 (1)	3.529 (4)	167 (1)
